# Neuroscience prospective on education

**DOI:** 10.3325/cmj.2014.55.428

**Published:** 2014-08

**Authors:** Lukasz M. Konopka

**Affiliations:** Department of Psychiatry, Loyola Medical Center, Maywood, IL, USA and Yellowbrick Consultation and Treatment Center, Evanston, IL, USA

As society evolves and makes new demands, how do we educate and nurture creative, free-thinking citizens for our increasingly complex world? Is our educational system truly preparing our youth for the future challenges to civilization, culture, and humanity? If we look at our current educational data, the answer seems to say, “Perhaps not.” So where are we failing? What are the issues we face in today’s education?

In today’s world, educators must compete with a myriad of electronic devices. Students are distracted and tantalized with immediate, powerfully stimulating images of graphic sex and violence, enhanced glamour, and gossip about the lives of the rich and famous. These data capture the brain’s attention like candy whetting the appetites of its primitive and most basic systems (1). On the other hand, our educational systems appeal to our higher rational brain. Does reason even stand a chance? Ultimately, how do we convince our youth that feeding the rational mind has as much appeal as Lady Gaga, six-pack abs, or Death Metal? Even in academic settings, students easily find answers to questions posed by educators by a quick browse through the internet (2). So, they find plentiful data, but they find them with little effort, thought, or consideration for how to interpret their sources.

We know that search engines prioritize and optimize the visibility and availability of data. Items that top their lists are viewed as the most significant and are used most frequently. From its design and purpose, the internet cannot ensure the integrity of its data. So how do students ensure the accuracy of a source? Generally, students assume that the internet’s public information meets reliable standards for truthfulness and undergoes rigorous scrutiny. They seem unaware that the internet has caused a tremendous information explosion that makes it very difficult to track and verify the vast quantity of public information. This explosion has buried our world in data. Therefore, our educational system must respond and begin significant restructuring of the learning process. Our youth must learn how to evaluate data, think critically, and filter irrelevant, misleading information from reliable worthy content (3).

Our political systems are examples of this data overload: Politicians often make statements that directly contradict their opponents. Although their audience, the general public, has the responsibility for verifying the politicians’ statements, veracity remains elusive. We don’t require politicians, their spokespeople, and media to account for their statements. Too often to further an agenda, everything and anything goes. Consequently, we, the public, are quite easily deceived. We rely on our primitive limbic brains and make decisions based on personal emotions that surround racial, political, and religious preferences. Thus, the political winners are those who shout the loudest, present the most emotionally compelling case, and gain the greatest funding and support from the most popular, visible individuals. If our future is data-driven and one where standards are moving targets and rational arguments are almost impossible to find, how do we prepare our youth? How will they develop the good judgment to make the best decisions? One solution may be through education that is geared toward critical thinking, keen skepticism for popular trends, and the creation of logical, databased, defensible arguments (4).

Such an education requires that when we train students, we design their education around an understanding of child development and the bio-psycho-social and spiritual processes. We must embrace the individual and individuality. We must not assume that all children learn the same way and fit into convenient algorithms. For many years, the educational system has attempted to create universal programs, and although this approach seems logical and economical, it is far from effective. Today, the US government, facing numerous educational failures, has developed the Core Curriculum, which proposes to address the needs of all students (5). The Core Curriculum assumes children have homogeneous gifts and weaknesses and, thus, applies one standard for all students. Yet, we are learning that this is absolutely the wrong approach. For example, within diverse populations we have failed to develop educational programs that enhance childhood learning in spite of attempts to individualize (6). When children are raised in poverty, are nutritionally compromised, have unstable parental structures, and lack environmental and social stability, it is unreasonable to expect them to learn the same ways as children who come from more enriched backgrounds. That is not to say that children from underprivileged environments do not learn and cannot succeed, but very different backgrounds require different approaches and may need to focus on different issues. Moreover, children from the well-to-do families also represent a varied population. Multiple factors cause the suffering of children: genetic predisposition, biological development, and environment. For example, we know of one young child who was raised in a safe, stable, loving, well-to-do family and experienced difficulty with language acquisition. From an early age, he could not learn the alphabet, had difficulty putting words together, and was unable to read. Therefore in primary school, as a consequence of his deficits, this child was severely ostracized and developed very poor self-concept, which led to his withdrawal, isolation, and significant shame. Later, his decreased self-worth carried into young adulthood, where he developed self-loathing and withdrew into a world of isolation and substance abuse. From his background, one might have anticipated a normal developmental trajectory, yet this young man was forced to re-establish his self-identity and struggled to re-enter the world as an independent adult. From a neurobiological standpoint, despite his auditory processing deficits, he had significant visual strengths and primarily interpreted the world through vision. Not surprisingly, because of his visual strengths, this individual spent significant time playing video games thriving on their visual input and immediate rewards. Unfortunately, his visual fascination led him into further isolation. So if we had understood his learning style when he was young, we could have intervened in his developmental course; instead, we must now attempt to reverse the negative processes that defined him, avoid the developmental difficulties that derailed his life, and provide an education that capitalizes on his visual strengths and avoids his verbal weaknesses. Although estimates are scarce for how many individual’s sensory processing issues leave them feeling isolated and drawn to aberrant behaviors such as addictions, we know the cases are numerous. Often these people are anxious, depressed, and eventually develop clinically significant psychiatric presentations.

How will these ideas influence our classrooms, our teachers, and our general educational system? What type of individuals become teachers? Are there gender biases in education? How do we train our teachers? Often our educational training programs ignore children’s neurobiological development and fail to recognize or understand their individuality. In the US, the students, who choose careers in education, are generally less motivated and less talented. In part, this situation is driven by career trajectories and economic potential. Frequently, our system under-pays, over-works, and under-appreciates our teachers giving them questionable social status. As a result, teachers become standardized, subsidized, babysitting services rather than truly transformative professionals. Generally, teachers graduate when they are relatively young and lack practical experience within their specializations. For example, math teachers often have little experience using mathematics in the workplace and opt to educate directly from textbooks. Not only does this approach limit a student’s mathematical understanding, it also ignores future real-world applications. Similarly, language teachers, who are deficient in language skills, cannot impart the subtle insights of how language impacts the future. Therefore, how can teachers teach without thorough understanding of their subjects and its utility in the workplace? Students would greatly benefit from teachers with real-world, hands-on experience. Outside the classroom, let’s give teachers fellowships or work experience so that they may share the excitement and practical utility of their specializations. In addition, let’s help teachers understand the cognitive neuroscience data behind the brain’s developmental stages (7). This awareness will guide their teaching so they know what can be expected from their students. When educators give students projects significantly below their ability, out of boredom, students become discouraged, unmotivated, and avoidant. Likewise, when students are challenged beyond their abilities, they may become anxious, scared, and defeated. There is a very delicate balance between providing exciting exploratory opportunities and overwhelming or boring the students. Therefore to enhance our educational systems, we must carefully select and organize students into categories of similar interest and neurocognitive development.

Happily, neuroscience can now help develop educational concepts that provide significant hope for individual students and the general educational population (8). Neuroscience’s discoveries augment our understanding of developmental trajectories (9,10), personal strengths and weaknesses, and suggest how we can adjust the process of education. Clearly, we are foolish to think of education as a hammer and every student as a nail. Recent data argue that significant genetic predispositions can enlighten our strategies for curricula design and student-centered education. We also understand that biological age may not predict the cognitive and emotional developmental stage of students. For instance, gender differences indicate that boys’ and girls’ emotional and cognitive skill sets differ during the various stages of development; therefore, our educational system must not only address cognition but also the emotions and social development of students (11). To assume that students are homogeneous throughout their education may lead to sub-optimal educational performance. When we acknowledge and embrace individuality and focus on emotional, social and cognitive development, we lay the groundwork for success in education.
